# Remarks upon Inflammation as Connected with Dental Surgery

**Published:** 1858-04

**Authors:** F. D. Thurman

**Affiliations:** Dentist, Atlanta, Georgia


### ARTICLE II.

*Remarks upon Inflammation as connected with Dental Surgery.* By F. D. Thurman, Dentist, Atlanta, Georgia.

No Diseased action, state or condition of the human system, is of more importance in its tendencies or consequences, than that of inflammation. In fact it is almost a universal attendant in a greater or less degree upon all diseased action. But its symptoms are more strikingly marked in diseases of a local nature. It is characterised by redness, swelling, heat and pain.

The theory of inflammation is not as yet very satisfactorily understood; we have the ingenious constructions of many learned men, though no two seem perfectly to agree. The distension of the capillaries, and increased quantum of blood in the part, is thought by many to be dependent on a want of action in the circulation, whilst others (equally eminent, and I

think with more reason,) conclude, that the blood is attracted by an increased action or stimulus of the part; whilst the general mass of Physicians are content to lay aside all theory on the subject, and treat each individual case acccording to the notion that happens to strike them when it is presented.

It is plain to be seen, that these conflicting theories require exactly reverse treatments, and when we take into consideration the important fact, that nearly every case that requires medical assistance is attended with more or less inflammatory action, it certainly seems that the subject demands more attention than Pathologists have bestowed on it.

But to the subject. Inflammation seems to be a wise provision of nature, set up for the purpose of relieving the system of obstructions, and is consequently always the effect and never the cause of the disease which it accompanies. Though a high state of inflammation may, and often does, by prostrating nervous influence, produce corresponding disease, and consequent inflammation of other parts.

Inflammation is certainly a friend to nature, and not an enemy; though it is a friend that requires watching, or it sometimes operates faster than the nervous system is willing, or even able to bear.

By the aid of inflammation, and its termination in suppuration, a foreign body may be removed from almost any part of the system, and cast off at the surface, and during the performance of this astonishing service, this very friend of nature, if suffered to operate too rapidly, will seriously involve other organs, or parts of the system in disease. Though this does not often happen, owing to the fact that nature in her wise provisions seems to act in inverse proportion—that is, she excites a high state of inflammation, and works rapidly in removing small obstructions, but when she attacks a more formidable enemy, she takes more time to prepare for the contest, and then passes quietly and cautiously to a termination. Just so with general or local disease of any kind. In acute diseases, the inflammatory action runs high, and a crisis is soon formed, whilst in chronic cases, the arterial excitement seems to be small in proportion to the small prospect of the patients final recovery. For two striking examples, we will mention Pneumonia and Consumption.

As soon as a part becomes diseased or obstructed, the blood rushes to its relief, and the pain consequent upon this state of things seems to be owing principally to the mechanical distention and consequent pressure upon the delicate nerves of the part. The more confined the parts are, the greater the pain. For instance, the Dental pulp, owing to its close confinement in the bony walls of the tooth, causes most excruciating pain, whilst the gums from the presence of salivary calculus, or old roots of teeth, may remain in a state of chronic inflammation for years, without attracting much attention. No part of the human system is more subject to inflammatory action, than the parts composing the mouth, and this is not surprising, when we take into consideration its numerous and complicated functions, to-wit: speech, prehension, mastication, insalivation, taste, inhalation, expiration, and the numerous and delicately constructed organs necessary to perform these various functions. And in addition to this, the mouth is the seat of the wonderful process of producing two sets of teeth. The first being removed by a gradual absorption of their roots, give place to the second set, which seem to be designed to last through life; but owing to causes too numerous to mention here; they seldom last to very old age. Though they may escape the ravages of decomposition, the economy often treats them as foreign bodies, on account of the salivary calculus or tartar which collects about their necks. All parts of the mouth are subject to inflammation, but I shall only notice at present, that form by which nature rids herself of diseased teeth.

As soon as a tooth or root of a tooth becomes so far diseased, that its deleterious effects are greater than its beneficial, the economy as noticed above, sets to work to get rid of it, but unfortunately this seems to be a very difficult task. No other foreign body seems to be so difficult to dislodge by the process of inflammation and suppuration, as a diseased tooth. This seems to be owing to the want of a sufficiency of fleshy or vascular substance to operate with. The root is only surrounded by a thin periosteal tissue which separates it from the bony walls of the alveolar processes, and notwithstanding this membrane becomes inflamed, and suppurates until it destroys itself, and keeps the root continually bathed in pus, its mechanical position keeps it in place for years, untilfinally after the periosteal tissue is so far destroyed that it can do nothing more, the absorbents on the exterior surface of the alveolus come to its assistance, and actually take down its surrounding bony wall, and leave the tooth or root to float in the gums; after which, it is soon removed by a tooth-pick, the patient's finger, or some other simple means. This process generally occupies from five to fifty years, but if the patient lives long enough, it will most assuredly be accomplished.

I will let these few remarks suffice for the present, though I have not by any means exhausted the subject'

